# Supplementary figures and images for: Worse than nothing at all: the inequality of fusions joining autosomes to the PAR and non-PAR portions of sex chromosomes
Source: PeerJ. 2024 Jul 23;12:e17740. doi: 10.7717/peerj.17740 (PMC11276758; doi:10.7717/peerj.17740)

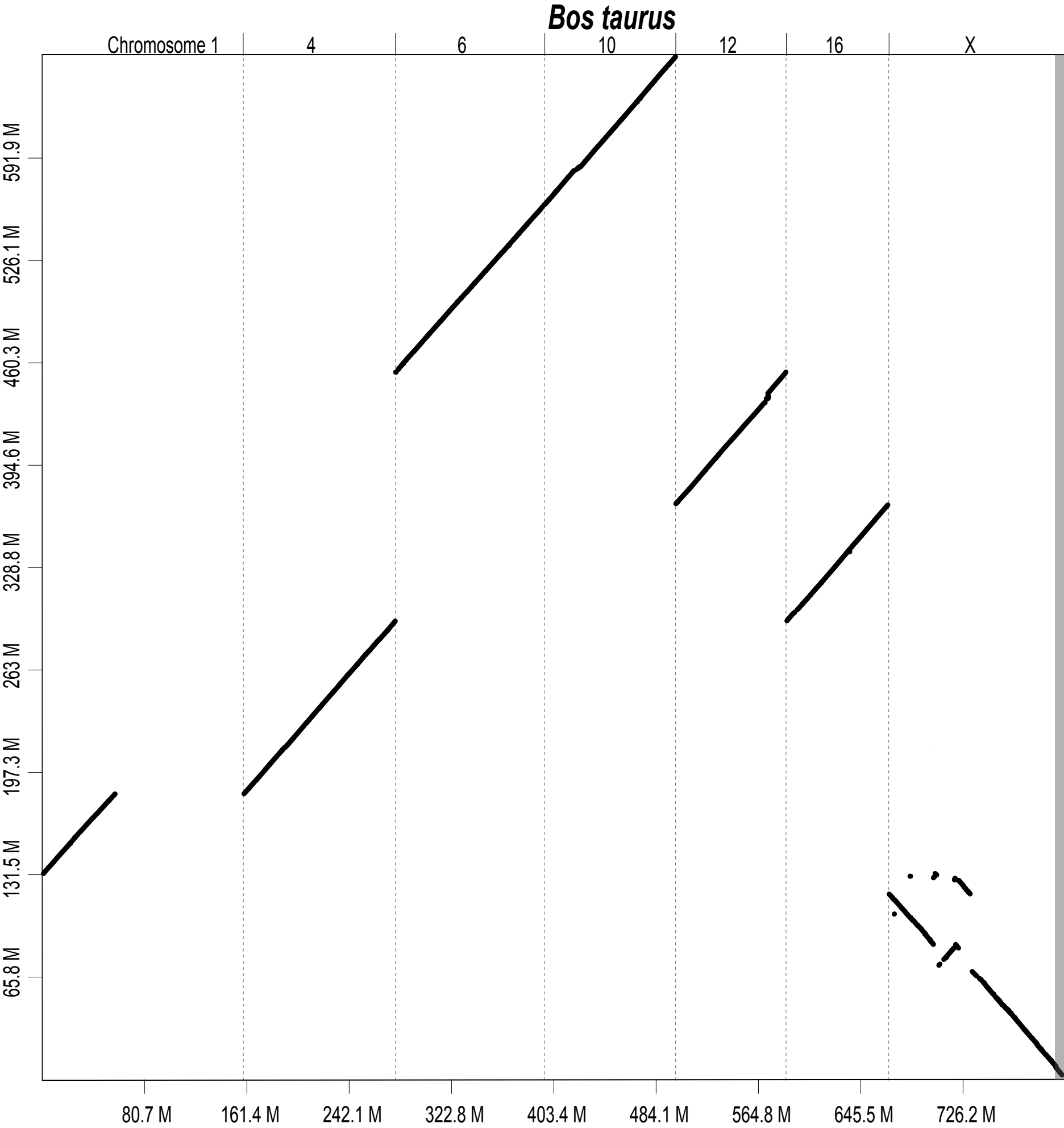

***Muntiacus muntjak***

Chromosome 3 + X

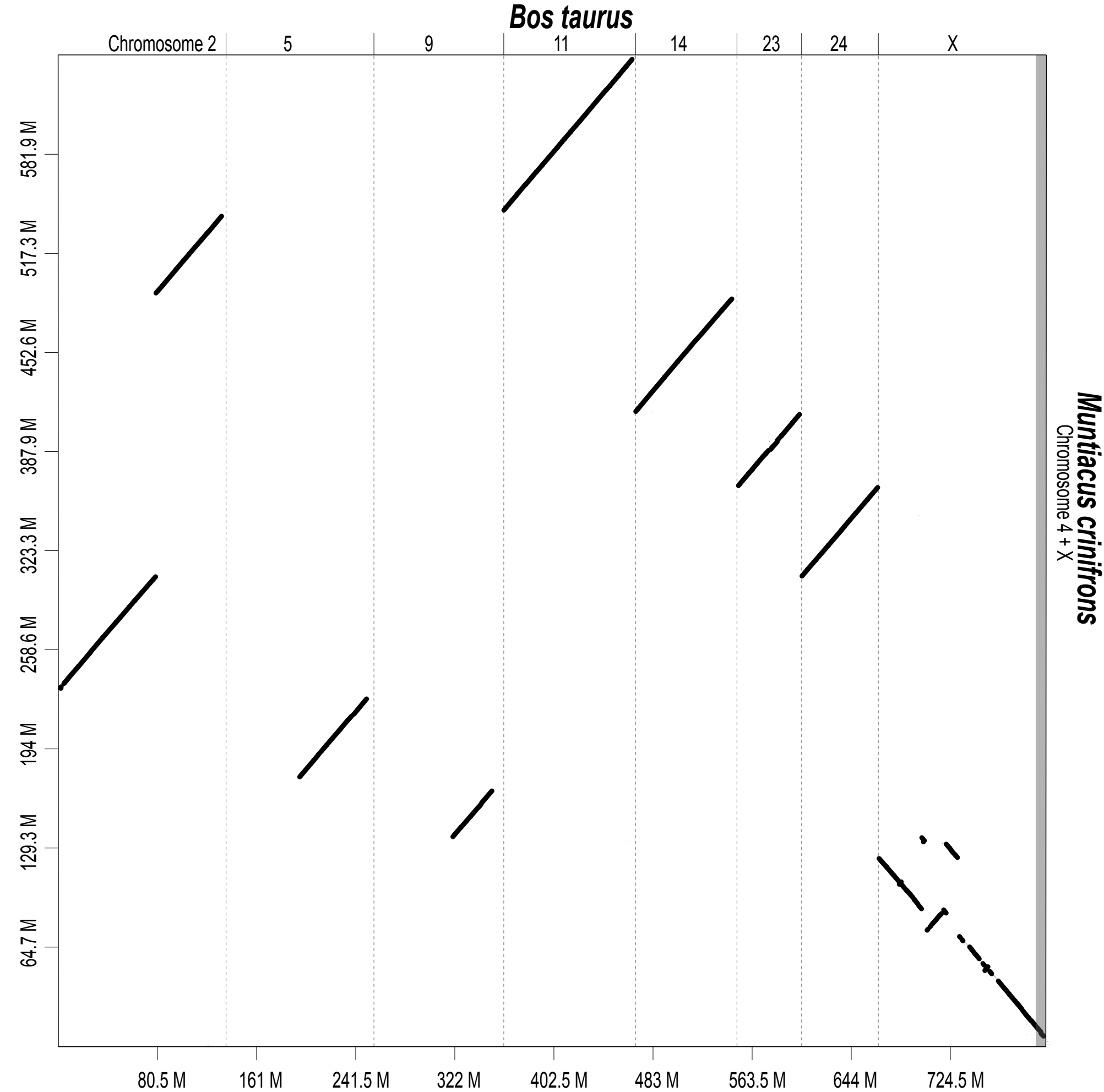

Supplement: Figure S1 — Dot-plot comparison of the fused autosome and X chromosome of two Muntiacus spp. against the Bos taurus autosomes & X chromosome. The x-axis represents Bos taurus while the y-axis is Muntiacus crinifrons (left) or Muntiacus muntjak (right). The black diagonal lines represent the aligned chromosomes, and the shaded gray region represents the PAR of Bos taurus. [file peerj-12-17740-s001.pdf]
